# Investigating lung responses with functional hyperpolarized xenon‐129 MRI in an ex vivo rat model of asthma

**DOI:** 10.1002/mrm.26003

**Published:** 2015-10-28

**Authors:** David M.L. Lilburn, Amanda L. Tatler, Joseph S. Six, Clémentine Lesbats, Anthony Habgood, Joanne Porte, Theodore Hughes‐Riley, Dominick E. Shaw, Gisli Jenkins, Thomas Meersmann

**Affiliations:** ^1^ Sir Peter Mansfield Imaging Centre, School of Medicine, University of NottinghamNottinghamUnited Kingdom; ^2^ Division of Respiratory MedicineNottingham University Hospitals, City Campus, University of NottinghamNottinghamUnited Kingdom; ^3^ Present address: Clinical Research Imaging Centre, Queen's Medical Research Institute, Little France Crescent, University of EdinburghEdinburghEH16 4TJUnited Kingdom; ^4^ Present address: Carestream Health Inc.8124 Pacific AvenueWhite CityOregon97503; ^5^ Present address: School of Science and Technology, Nottingham Trent University, Clifton CampusClifton LaneNottinghamNG11 8NSUnited Kingdom

**Keywords:** pulmonary imaging, hyperpolarized noble gas MRI, hp ^129^Xe, ovalbumin (OVA) rat model of asthma, methacholine challenges, airway hyper‐responsiveness

## Abstract

**Purpose:**

Asthma is a disease of increasing worldwide importance that calls for new investigative methods. Ex vivo lung tissue is being increasingly used to study functional respiratory parameters independent of confounding systemic considerations but also to reduce animal numbers and associated research costs. In this work, a straightforward laboratory method is advanced to probe dynamic changes in gas inhalation patterns by using an ex vivo small animal ovalbumin (OVA) model of human asthma.

**Methods:**

Hyperpolarized (hp) ^129^Xe was actively inhaled by the excised lungs exposed to a constant pressure differential that mimicked negative pleural cavity pressure. The method enabled hp ^129^Xe MRI of airway responsiveness to intravenous methacholine (MCh) and airway challenge reversal through salbutamol.

**Results:**

Significant differences were demonstrated between control and OVA challenged animals on global lung hp ^129^Xe gas inhalation with *P* < 0.05 at MCh dosages above 460 μg. Spatial mapping of the regional hp gas distribution revealed an approximately three‐fold increase in heterogeneity for the asthma model organs.

**Conclusion:**

The experimental results from this proof of concept work suggest that the ex vivo hp noble gas imaging arrangement and the applied image analysis methodology may be useful as an adjunct to current diagnostic techniques. Magn Reson Med 76:1224–1235, 2016. © 2015 The Authors. Magnetic Resonance in Medicine published by Wiley Periodicals, Inc. on behalf of International Society for Magnetic Resonance in Medicine. This is an open access article under the terms of the Creative Commons Attribution License, which permits use, distribution and reproduction in any medium, provided the original work is properly cited.

## INTRODUCTION

New insights into the pathophysiology of asthma, a chronic inflammatory disorder of the airways 1, have been provided over the last 20–30 years by a variety of molecular and biomedical techniques 2, 3, some of which, such as monoclonal antibody technologies 4, have resulted in new treatments. Unfortunately, despite this progress, rates of asthma continue to rise with the disease becoming of increasing importance worldwide 5, 6, 7. The advancement of new methods that provide a better understanding of the disease and that facilitate drug development studies is, therefore, urgently required.

Rodent models of asthma are widely used to study the disease 8, 9 and have allowed for advances in the understanding and treatment of the condition. For example, the development of inhaled corticosteroid and bronchodilator medications and the need for combination therapies have resulted from such studies 10, 11, 12, 13. There are several animal models of asthma currently in use that display many features of human asthma 14, 15, 16, 17 with the ovalbumin (OVA) model of allergic asthma one of the best characterized 18. Although rodent responses include high IgE titers, similar to human disease, 19, 20, levels of airway hyper‐responsiveness (AHR) are often low when measured by conventional respiratory measurements 18, 21. The imaging methodologies of ultrashort echo time (UTE) MRI and microcomputed tomography have since been used to study airway inflammation 22 and remodeling 23, respectively. Hyperpolarized (hp) noble gas MRI, however, has the ability to provide information on lung responses with direct visualization of regional changes in ventilation 24, 25, 26, 27 potentially allowing for the sensitive detection of AHR.

There has been a move toward the use of ex vivo lung tissue to investigate lung responses in health and disease in an effort to limit research costs and impact the “3Rs” of ***r***educing animal numbers, ***r***eplacing animal experiments by alternative techniques or ***r***efining procedures to limit animal suffering 28, 29. An additional benefit of ex vivo experiments is the ability to study physiological and functional respiratory parameters independent of confounding systemic variables. Examples of such variables include: alterations in respiratory rate and volumes; anesthetic considerations, coupled with the effects of bronchoactive substances on other organ systems; and nonpulmonary compensation mechanisms 28, 30, 31. In light of this, precision‐cut lung slice (PCLS) models in particular are gaining increasing acceptance as a method to study lung responses 32, 33, 34, 35, 36, 37. While there is much benefit to be derived from PCLS models there are notable problems in terms of the removal of the intact circulation and bronchial tree, resulting in a nonphysiological delivery of compounds to the lung 28.

As an alternative to the PCLS methodology, whole organ ex vivo models may be used 30, 31, 38, benefiting from the absence of systemic influences while retaining delivery methods for compounds more akin to the in vivo situation 39, 40 and allowing for the study of whole organ factors such as hyperventilation 41. Furthermore, previous work has explored airway responsiveness in the ex vivo mouse lung using conventional respiratory measurements 42. The purpose of this work was, therefore, to investigate differences in gas inhalation patterns during repeated intravenous challenges with the muscarinic agonist, methacholine, using the previously developed ex vivo hp ^129^Xe MRI methodology 43.

## METHODS

### Model Characterization and Preparation for Ex Vivo hp ^129^Xe MRI

#### Animals

The University of Nottingham Ethical Review Committee approved the study, which was carried out in accordance with the UK Home Office Animals (Scientific Procedures) Act 1986. Male Brown‐Norway rats (150–199 g; n = 18) were purchased from Charles River Ltd (Margate, UK) and housed in the Biomedical Services Unit, University of Nottingham for at least 7 days before commencement of the study. Animals had free access to food and water.

#### Ovalbumin Induced Asthma

**Figure 1:**
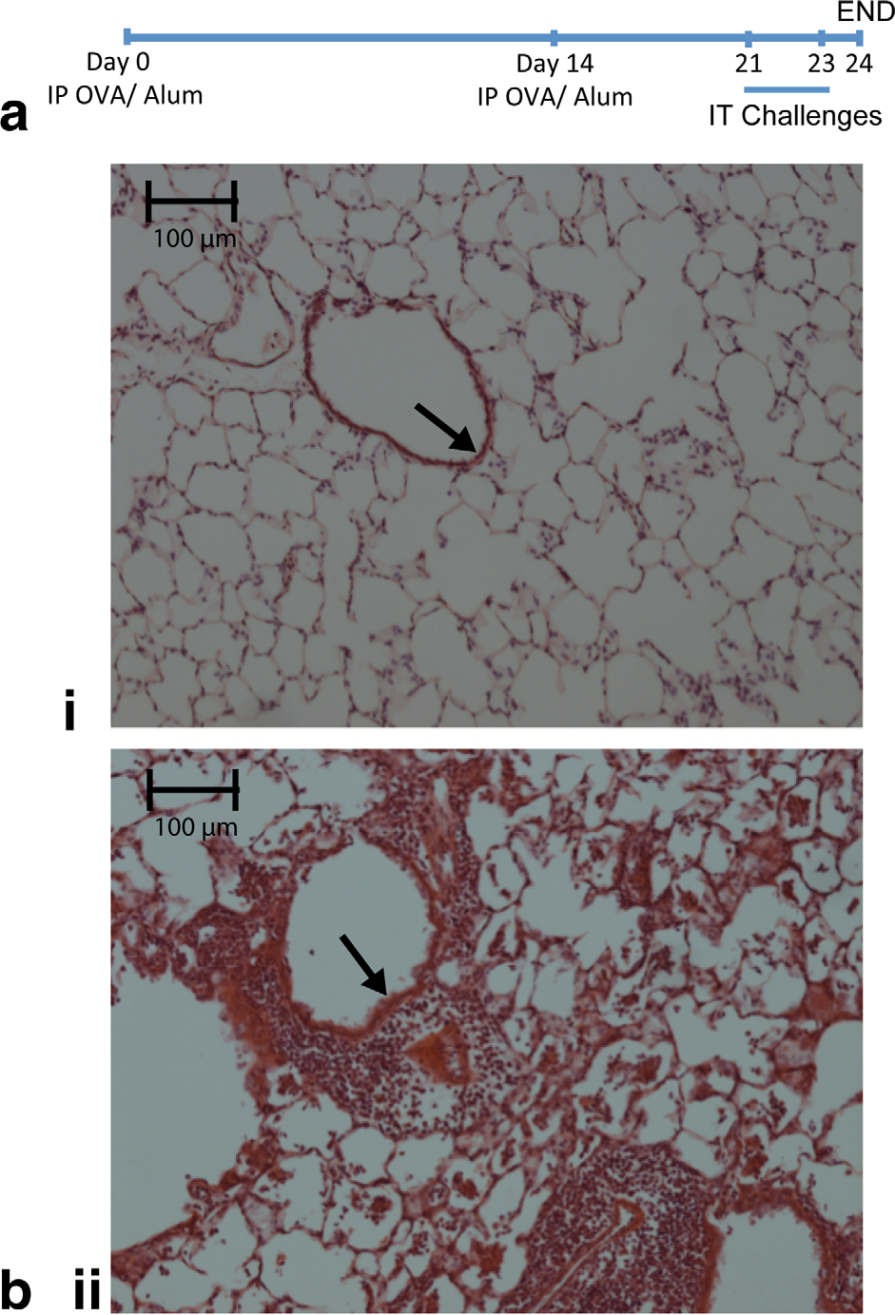
Sensitization and challenge protocol with typical light microscopy images of control and OVA challenged lungs. **a**: All rats were sensitized with intrapertioneal (IP) injections of 2 mg of ovalbumin and 200 μg of aluminum hydroxide (Alum) on day 0 and day 14 with subsequent intratracheal (IT) challenges on days 21 and 23 of either 300 μL of sterile PBS (control animals) or 300 μL of OVA (500 μg/250 μg of OVA) with the same OVA dose on each challenge. Animals were killed on day 24 for either BAL/histology or ex vivo hp ^129^Xe MRI. **b**: Light microscopy image from (i) control (PBS challenged) lung and (ii) 500‐μg OVA challenged lung. Note the clear airspaces and thin bronchial walls of the large airways (arrow) in the control lung in contrast to the marked increase in cellular infiltrate particularly around the large airways (arrow) in the OVA challenged lung.

Animals were sensitized by intraperitoneal injections of 2 mg of ovalbumin (OVA) with 200 μg of aluminum hydroxide (Alum) in 1 mL on day 0 and day 14 44 (see Figure 1a). Animals were challenged with increasing concentrations of intratracheal (i.t.) OVA in 300 μL of sterile phosphate buffered saline (PBS, Sigma‐Aldrich Ltd, Gillingham, UK), or PBS only control, on days 21 and 23, administered under inhaled general anesthesia (4% isofluorane 96% medical grade oxygen at a flow rate of 2L/min for 3–4 min). On day 24, animals were weighed and euthanized by overdose of sodium pentobarbital (Sigma‐Aldrich Ltd, Gillingham, UK).

#### Preparation for Ex Vivo hp ^129^Xe MRI

Lungs for ex vivo hp ^129^Xe imaging had a cannula inserted into the caudal vena cava to allow flushing of the pulmonary circulation with heparin 100 IU/mL (Wockhardt UK Ltd, Wrexham, UK) in 20 mL 0.9% saline solution followed by 20 mL of PBS to remove residual blood from the pulmonary circulation. The heart and lungs were removed en masse. A polytetrafluorethylene (PTFE) adapter tube was inserted 5–10 mm above the carina and sutured into place. The heart and lungs were suspended in Hartmann's solution (Baxter Healthcare Ltd, Thetford, UK) in the custom‐built ventilation chamber with the trachea pointing downward as detailed previously 43. The ex vivo lungs were repeatedly inflated with 5–6 mL of room air to check for gas leaks. The lungs were chilled to 278 K for transportation to the imaging facility. After transportation, the lungs were passively warmed to ambient temperature before imaging. The time from harvest to MR imaging was no more than 5 h.

Of all animals enrolled in the study, six rats were treated with 500 μg OVA i.t., three rats were treated with 250 μg OVA i.t. and nine control rats were treated using i.t. D‐PBS. Of these animals, three 500 μg OVA and three D‐PBS control rats were used solely for BAL and histological analysis. During the hp ^129^Xe experiments, one 250‐μg OVA treated rat lung proved unsuitable for imaging due to gas leakage with three control lungs suffering from similar issues, therefore, also being rejected for ex vivo hp ^129^Xe MRI.

#### Histology

Lungs were inflated with 10% formalin‐saline solution (Sigma‐Aldrich Ltd, Gillingham, UK) at a pressure of 25 cm of H_2_O (≈ 2.5 kPa) for 24–48 h and paraffin‐wax embedded. Five‐micron sections were stained with hematoxylin and eosin (H&E). The resulting H&E stained histological sections were visualized using a Nikon Eclipse 90i microscope (Nikon Corporation, Tokyo, Japan) with NIS Elements Ar image analysis software for image capture (v.3.2, Nikon Corporation, Tokyo, Japan).

#### Bronchoalveolar Lavage

A separate group of control and OVA challenged rats that were not subjected to MR imaging had bronchoalveolar lavage (BAL) performed post mortem with 1 mL of PBS instilled i.t. before subsequent removal. BAL samples were centrifuged at 231 × g (1500 rpm) for 5 min with the resulting cell pellet resuspended in 1 mL of PBS. Total BAL cell count was performed with a Sceptor automated cell counter (Millipore, UK). BAL cells were then centrifuged at 100 × g onto slides and stained using Diff Quik Romanowski stain (Fisher Scientific, UK). The % of differential inflammatory cells was determined by counting 100 cells using a light microscope at 40× magnification.

### ^129^Xe spin exchange optical pumping, compression and transfer

Hp ^129^Xe was produced in batch mode using spin exchange optical pumping (SEOP) 45, 46 of a gas mixture containing 25% Xe (enriched to 83% ^129^Xe, Nova Gas Technologies, Charleston, SC, USA) and 75% nitrogen (N_2_, 99.999% pure, Air Liquide, Coleshill, UK). SEOP was performed at 40–60 kPa followed by hp gas delivery to the lung as reported in ref. 47. The hp Xenon was not cryogenically separated before delivery to the excised lungs for inhalation. The spin polarization was approximately *P* = 40% that, factoring in the four‐fold gas dilution, provided an “apparent” spin polarization of *P_app_* = 10% 48.

### Hp Gas Inhalation

The ventilation chamber with the ex vivo lungs was placed inside the bore of the superconducting magnet and held at a constant temperature of 293 K. Active inflation of the lung was accomplished by pulling to a ventilation syringe volume (*V_s_*) of 6 mL. Corresponding inhaled volumes (*V_i_*) were measured separately using a water displacement technique on gas exhalation listed in Table 1. To limit potential gas trapping, the ex vivo lungs were deflated over 30–60 s from *V_s_* = 6 mL to maximum exhalation (*V_s_* = 0 mL) as reported elsewhere 49, 50 before hp ^129^Xe inhalation.

**Table 1:** Demographic Data from Subjects Used for hp ^129^Xe Imaging^a^

	Identifier	OVA challenge dose (μg)	Rat weight (g)	Inhaled volume *V_i_* (mL)
CONTROL	C.1	N/A	259	4.2 ± 0.3
	C.2	N/A	262	5.0 ± 0.4
	C.3	N/A	218	5.2 ± 0.4
OVA CHALLENGED	OVA.1	500	252	4.9 ± 0.3
	OVA.2	500	254	4.9 ± 0.4
	OVA.3	500	270	4.6 ± 0.1
	OVA.4	250	223	5.1 ± 0.1
	OVA.5	250	210	4.8 ± 0.1

Summary of rat weights at the time of organ harvest, ovalbumin (OVA) dosages, and inhaled volumes (*V_i_*) ± standard deviation corresponding to inflation (syringe) volume *V_s_* = 6 mL. Note all control animals received 300 μL sterile D‐PBS on airway challenges.

### Bronchoconstriction and Reversal

Animals used for hp ^129^Xe MRI experiments had the catheter used for flushing of the pulmonary circulation retained. The cranial vena cava was ligated to ensure drug delivery to the pulmonary circulation. The cannula in the caudal vena cava was sutured into place and attached to 1.6‐mm outer diameter perfluoroalkoxy (PFA) tubing for administration of drugs to the pulmonary circulation as detailed in previous work 43. Excess fluid was removed to keep the fluid level within the ventilation chamber constant during the imaging experiments. In an attempt to satisfy tissue metabolic demands, the lungs were ventilated 8–10 times with the 100% oxygen immediately after imaging followed by purging the lungs and transfer line with N_2_ before MCh (with Hartmann's solution) and hp ^129^Xe delivery. This resulted in the lungs being held in an anoxic condition for less than 3–4 min.

Initial hp ^129^Xe MR imaging was repeatedly performed at baseline to ensure reproducibility of hp gas inhalation with multiple images (minimum of two) acquired and visually inspected to ensure a stable reference point for comparison of later image data on increasing MCh dosages. Furthermore, 4.5 mL of Hartmann's solution was delivered at a rate of 1–2 mL/min before bronchoprovocative challenges to ensure there were no significant changes on hp ^129^Xe MRI after fluid administration to the lung.

Bronchoconstriction was achieved by delivering methacholine (MCh, Sigma‐Aldrich Ltd, Gillingham, UK) through the pulmonary circulation as detailed previously 43. Increasing doses of 10, 25, 50, 75, 100, 200, and 400 μg of MCh dissolved in 1 mL of 0.9% saline solution followed by a 3.5‐mL bolus of Hartmann's solution at a rate of 1–2 mL/min were delivered before sequential hp ^129^Xe imaging experiments. Each image was acquired on a separate inhalation of hp ^129^Xe. The maximum cumulative dose of MCh delivered to each set of excised lungs was 860 μg. The bolus of Hartmann's solution following the MCh was to ensure complete drug delivery to the lung by flushing out the dead volume in the delivery system. Reversal of bronchoconstriction was attempted by flushing the lungs with 30–50 mL of Hartmann's solution and 1000–1500 μg of salbutamol (Allen and Hanbury's Ltd, Middlesex, UK) in 1.0–1.5 mL at a rate of 1–2 mL/min. The monitored airway pressure during inhalation was kept constant between successive images using the pressure value at baseline for an inflation syringe volume *V_s_* = 6 mL. Typical airway pressures recorded during inhalation in control and OVA challenged lungs were 2.2–2.6 kPa (22–26 cm H_2_O) and 2.5–3.0 kPa (25–30 cm H_2_O), respectively.

### MRI Protocol

MR imaging experiments were performed using a 9.4 Tesla (T) vertical bore Bruker Avance III microimaging system (Bruker Corporation, Billerica, MA). A custom‐built 25 mm low‐pass birdcage volume coil tuned to the resonance frequency of ^129^Xe gas (110.69 MHz) was used in all experiments. Images were acquired using a modified variable flip angle (VFA) FLASH gradient echo pulse sequence 51. Rectangular pulses of 134 μs (1.8 KHz bandwidth) and sinc‐shaped pulses of 1000 μs (240 Hz excitation bandwidth) at variable power levels were used for non–slice‐selective and slice‐selective image acquisitions (echo time = 1.27 ms; repetition time = 67.5 ms). All coronal images were acquired in 128 × 64 image matrices with a field of view (FOV) of 47.3 mm and 31.5 mm in the read and phase‐encoding directions, respectively. Total acquisition time was 4.38 s. At a frequency domain spectral bandwidth of 50 KHz, this relatively long acquisition period was solely due to limitations of the custom built coil to prevent coil heating and arcing. Note that the long acquisition time may have caused some diffusive attenuation and image blurring. Signal‐to‐noise ratio (SNR) values from non–slice‐selective hp ^129^Xe images were ∼70 and ∼50 at baseline in the healthy and OVA treated lungs, respectively. Slice‐selective imaging experiments were performed where a single 4 mm central slice was acquired with a resulting nominal resolution of 0.37 × 0.49 × 4 mm^3^. SNR values from the slice selective images were ∼30 and ∼20 for the healthy and OVA treated lungs, respectively.

### Image Reconstruction and Analysis

Images were processed and reconstructed in Prospa (v.3.06, Magritek, Wellington, New Zealand) with a sine‐bell squared window function applied to the raw data before two‐dimensional (2D) Fourier transformation. The 2D image data were then exported for analysis in IGOR Pro (v.6.01, Wavemetrics, Lake Oswego, OR). Calculation of whole lung SNR values were made by thresholding the image matrix with the lower threshold derived from the mean signal intensity plus three standard deviations obtained from a 10 × 10 voxel region randomly selected outside the lung region within the image limits.

### Measurement of Global Reduction in hp ^129^Xe Inhalation

As a measure of the reduction in global hp ^129^Xe signal across the whole lung, the normalized global signal intensity, GS , was obtained for all steps on the MCh challenge protocol (and after salbutamol reversal) by dividing the sum of all *k* voxel values $\sum_{i}^{i=k}M_{i}$ across each image by the sum of all *k* voxel values of the baseline image $\sum_{i}^{i=k}B_{i}$:

(1) $$GS=\sum_{i}^{i=k}M_{i}/\sum_{i}^{i=k}B_{i}$$

where the subscript *i* denotes the voxel number. To ensure that no fluctuation in the background noise would affect the normalized global signal intensity, the noise levels from 10 × 10 voxel regions far‐removed from the lung were monitored, with mean noise values calculated and compared between images in the MCh challenge sequence. Mean noise values were seen to be consistent between subsequent images with fluctuation of <20%.

### Visualization of Changes in Regional hp ^129^Xe Inhalation Caused by MCh Challenges and Reversal

Maps of absolute intensity difference, $\Delta A_{i}$ , were calculated by subtracting the baseline image data from data on increasing MCh dosages ( $M_{i}$) similar to the method reported by Mistry et al 52:

(2) $$\Delta A_{i}=M_{i}-B_{i}$$

In analogy, difference maps on reversal were calculated using image data of the 400 μg MCh dose (860 μg cumulative dose MCh) for subtraction from the image acquired on reversal.

Maps of relative intensity difference, $\Delta R_{i}$, were obtained by dividing each voxel value $M_{i}$ by the mean voxel intensity $k^{-1}\sum_{i}^{i=k}M_{i}$ resulting in the relative intensity data of the respective MCh challenge. The relative intensity data of the baseline image is then subtracted and the resulting expression is further scaled by the global signal intensity, GS:

(3) $${\Delta R}_{i}=\left(\frac{M_{i}}{k^{‐1}\sum_{i}^{i=k}M_{i}}‐\frac{B_{i}}{k^{‐1}\sum_{i}^{i=k}B_{i}}\right)\times \frac{100\%}{GS}$$

with *k* as the total number of voxels. In analogy, relative intensity difference maps for the reversal were obtained by subtracting the 400 μg dose of MCh (860 μg cumulative dose MCh) data from the salbutamol reversal data.

### Statistical Analysis

Statistical analysis was performed using GraphPad PRISM (v.6.0, GraphPad Software Inc., La Jolla, CA). Mean total inflammatory cell counts and differences in SNR values were compared using the student's t‐test. Bland‐Altman analysis was performed to compare % difference between the nonslice and slice‐selective values of normalized global signal intensity. Comparison of the dose response curves between the control and OVA challenged groups was performed using a two‐way analysis of variance with the Bonferroni test for post hoc comparison between cumulative MCh dosages (as compared with baseline measurements). Probability values *P* < 0.05 were deemed to be significant.

## RESULTS

### OVA Sensitized and Challenged Rats Develop Eosinophilic Airways Disease

**Figure 2:**
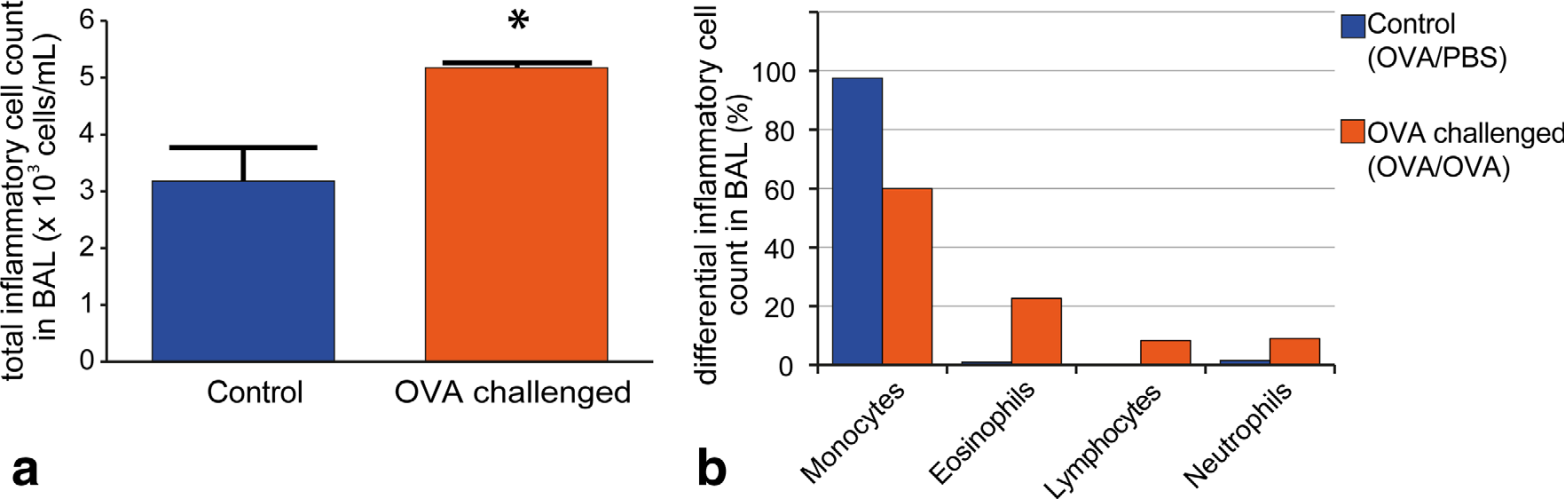
Bronchoalveolar lavage (BAL) data. % differential inflammatory cell counts. Data from satellite (histology) group with BAL performed immediately after death. **a**: Note the statistically (*P* = 0.03) higher inflammatory cell count in the OVA challenged group (OVA sensitized/OVA challenged) compared with the control group (OVA sensitized/PBS challenged). Similarly note the increased % differential eosinophil count in the OVA challenged group **b**: indicating allergic inflammation of the airways was present. Total inflammatory cell counts with standard deviations (error bars) are calculated from sample sizes n = 3 in both OVA challenged and control groups. The control BAL % differential cell count is calculated from a sample size n = 2 compared with n = 3 in the OVA challenged group.

Qualitative comparison of histological lung sections revealed notable thickening of the bronchial epithelium and accumulation of inflammatory cells in OVA challenged rats when compared with control animals (Fig. 1). Quantitative analysis of BAL inflammatory cells demonstrated that total inflammatory cells present in BAL samples were significantly increased in OVA challenged animals (*P* = 0.03; Figure 2a) and differential cellular analysis confirmed that the increase was predominantly in eosinophils (Fig. 2b) in line with previous studies 19, 53, 54.

### Dynamic Changes in hp ^129^Xe Gas Distribution on Increasing Dosages MCh and Salbutamol Reversal

**Figure 3:**
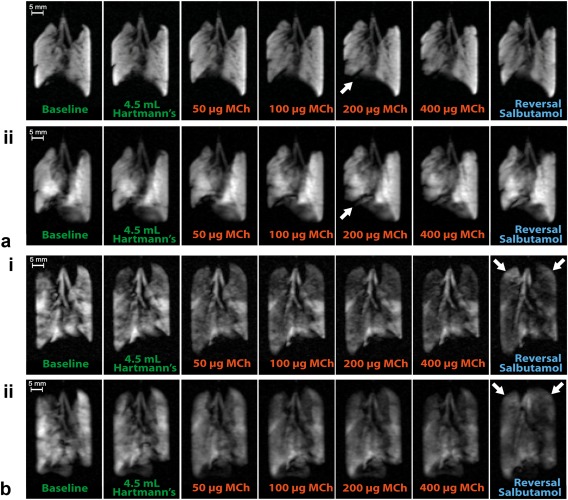
VFA FLASH image datasets from a) control lung and b) ovalbumin (OVA) treated lung at baseline, on perfusion with Hartmann's solution, and on increasing dosages of methacholine (MCh) with subsequent reversal. **a**: (i) Slice selective and (ii) nonslice selective image data from control rat C.1. There is a large ventilation defect in right lower lobe seen after 200 μg of MCh (white arrows) with subsequent reversal with salbutamol and Hartmann's solution. **b**: (i) Slice selective and (ii) nonslice selective image data from OVA challenged rat OVA.1. Due to on‐going inflammation within the lung there is significant heterogeneity in the hp ^129^Xe distribution on the baseline images. A small reduction in signal intensity is seen in the right cranial lobe after flushing with 4.5 mL of Hartmann's solution. Noticeable reduction in signal intensity is seen after the 50‐μg dose of MCh with further large reductions seen until 400 μg of MCh. Subsequent reversal with salbutamol and Hartmann's solution results in a slight increase in the hp ^129^Xe gas signal in the right cranial lobe and the upper portion of the left lobe (white arrows). The full image datasets for lung C.1 and OVA.1 with multiple baseline images and those acquired after each dose of MCh are displayed in the Appendix 1 in the Supporting Information.

Images at key points of the MCh challenge protocol from control and OVA challenged lungs demonstrating the differences in regional gas distribution and inhalation volume for increasing MCh doses are displayed in Figure 3 (full dataset in Appendix 1 in the Supporting Information, which is available online). The distribution of hp ^129^Xe is notably heterogeneous in the OVA challenged lungs even at baseline compared with the control lungs despite comparable inhalation volumes (Table 1). The mean lung SNR value is higher, although nonsignificantly different, in the control lungs versus the OVA treated group (76.6 ± 22.1 versus 51.7 ± 9.4, *P* = 0.15). Volume and distribution of hp ^129^Xe appears largely unchanged after a 4.5‐mL bolus of Hartmann's, although small changes became visible after the initial deliveries of fluid to the pulmonary circulation in OVA.1 (Fig. 3b) and control lung C.3. On increasing doses of MCh the presence of ventilation defects was noticeable in one control lung after 200 μg of MCh (460 μg cumulative MCh dose), one 250‐μg OVA challenged lung after 100–200 μg of MCh (260‐ to 460‐μg cumulative MCh dose), and in two 500‐μg OVA challenged lungs after 50–75 μg of MCh (85‐ to 160‐μg cumulative MCh dose). However, there were significant reductions in the overall signal intensities in all OVA challenged lungs compared with control lungs (mean whole lung SNR at 860‐μg cumulative MCh dose for the control lungs = 70.9 ± 28.0 versus 18.2 ± 1.7 for OVA treated lungs, *P* = 0.03). Some reversal in the gas volume reduction induced by the MCh challenge became apparent in the images after varying volumes of Hartmann's solution and up to 1500 μg of salbutamol in two control animals (C.1 and C.3) and three OVA challenged animals (OVA.1, OVA.4, and OVA.5).

### Global Changes in hp ^129^Xe Inhalation Caused by MCh Challenges

**Figure 4:**
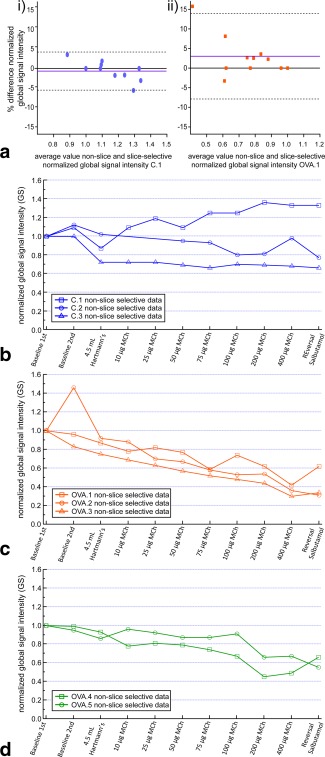
**a**: Bland‐Altman plots comparing nonslice and slice selective normalized global signal intensity data, GS (Eq. [1]), from the images in sequences (i) Figure 3a (rat lung C.1) and (ii) Figure 3b (rat lung OVA.1) normalized to the 1^st^ baseline values in each image sequence (as all other data in this figure). Dashed grey and solid purple horizontal lines represent the 95% limits of agreement and the mean bias, respectively. **b**: Normalized global signal intensity data from the nonslice selective images of hp ^129^Xe imaged control lungs. There is an initial drop in the normalized global signal intensity from the baseline images to the image after 10 μg of MCh in lung C.3, with minimal subsequent decreases (or increases as in the case of C.1) over the sequence. Note that the data points at 10 and 25 μg of MCh for C.2 were lost. **c,d**: Similarly acquired data as in (b) with (c) representing data from lungs challenged with 500 μg of OVA and (d) data from lungs challenged with 250 μg OVA. Note the marked reduction in normalized global signal intensity over the sequence of events in the OVA challenged lungs with a greater reduction in the lungs challenged with 500 μg of OVA.

The normalized global signal intensity, GS, (see Eq. [1]) was obtained from slice selective and nonslice selective measurements of control and OVA challenged lungs with the results used for a Bland‐Altman plot in Figure 4a. The determined bias was −0.65 ± 2.39% in the control lung (C.1) and 3.03 ± 5.57% in the OVA treated lung (OVA.1), suggesting little systematic difference between the techniques. The nonslice selective data were used for further comparison as this allowed for monitoring changes in whole lung hp gas inhalation (Figs. 4b–d).

**Figure 5:**
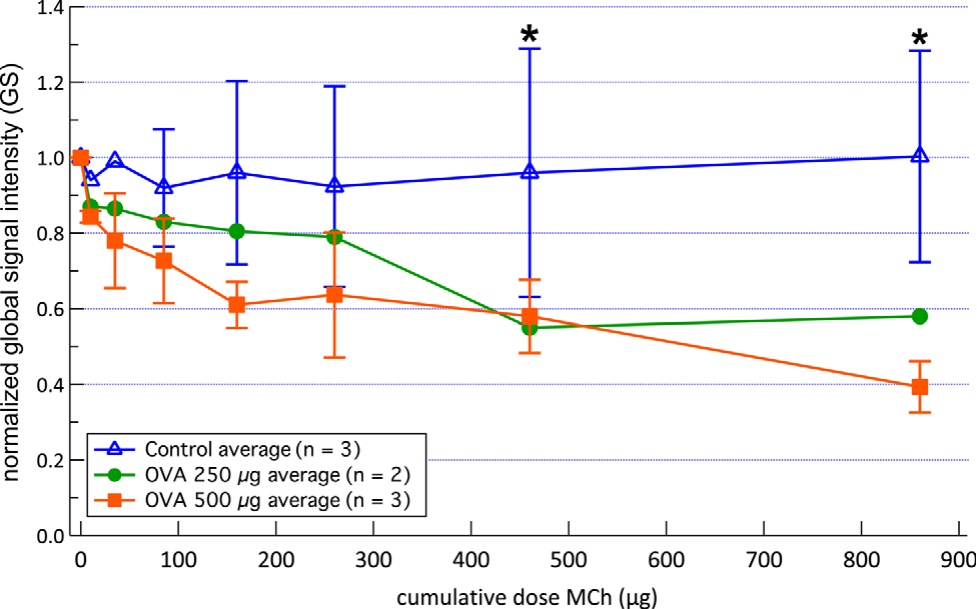
Normalized global signal intensity, GS, averaged over all control and OVA challenged groups as a function of cumulative dose of MCh. Values are normalized to the average value calculated from: the two baseline images, the image after 4.5 mL of Hartmann's solution, and the image after 10 μg of MCh (to compensate for the effect of small fluctuations in the SEOP process). Error bars represent ± standard deviation. Note that no standard deviations were available for the 250‐μg OVA challenged group and for the first two points of the control group (see text for detail). A significant difference (*p* < 0.0001) was detected on two‐way ANOVA of the dose responses between control and 500‐μg OVA challenged groups on the dose responses. Significant difference (* as indicated in figure) in response (*p* < 0.05) measured by a reduction in the normalized global signal intensity was found on cumulative MCh dosages above 460 μg for the 500‐μg OVA challenged group (*p* = 0.048 and *p* = 0.0015 at 460‐μg and 860‐μg cumulative dosages of MCh, respectively).

Values of the normalized global signal intensity, GS, averaged for all lungs in the control and OVA challenge groups are shown in Figure 5 as a function of cumulative MCh dose. In Figure 5, it can be seen that there is an initial significant drop in global hp gas inhalation between a cumulative dose of 10–160 μg MCh in the OVA challenged lungs followed by a slower reduction up to the maximum cumulative dose of 860 μg.

The 500‐μg OVA challenged group showed a significant reduction (*P* < 0.05) in the normalized global signal intensity over baseline at cumulative MCh dosages above 460 μg (see Figure 5 for details).

### Visualization of Changes in hp ^129^Xe Inhalation Patterns Caused by MCh Challenges and Reversal

**Figure 6:**
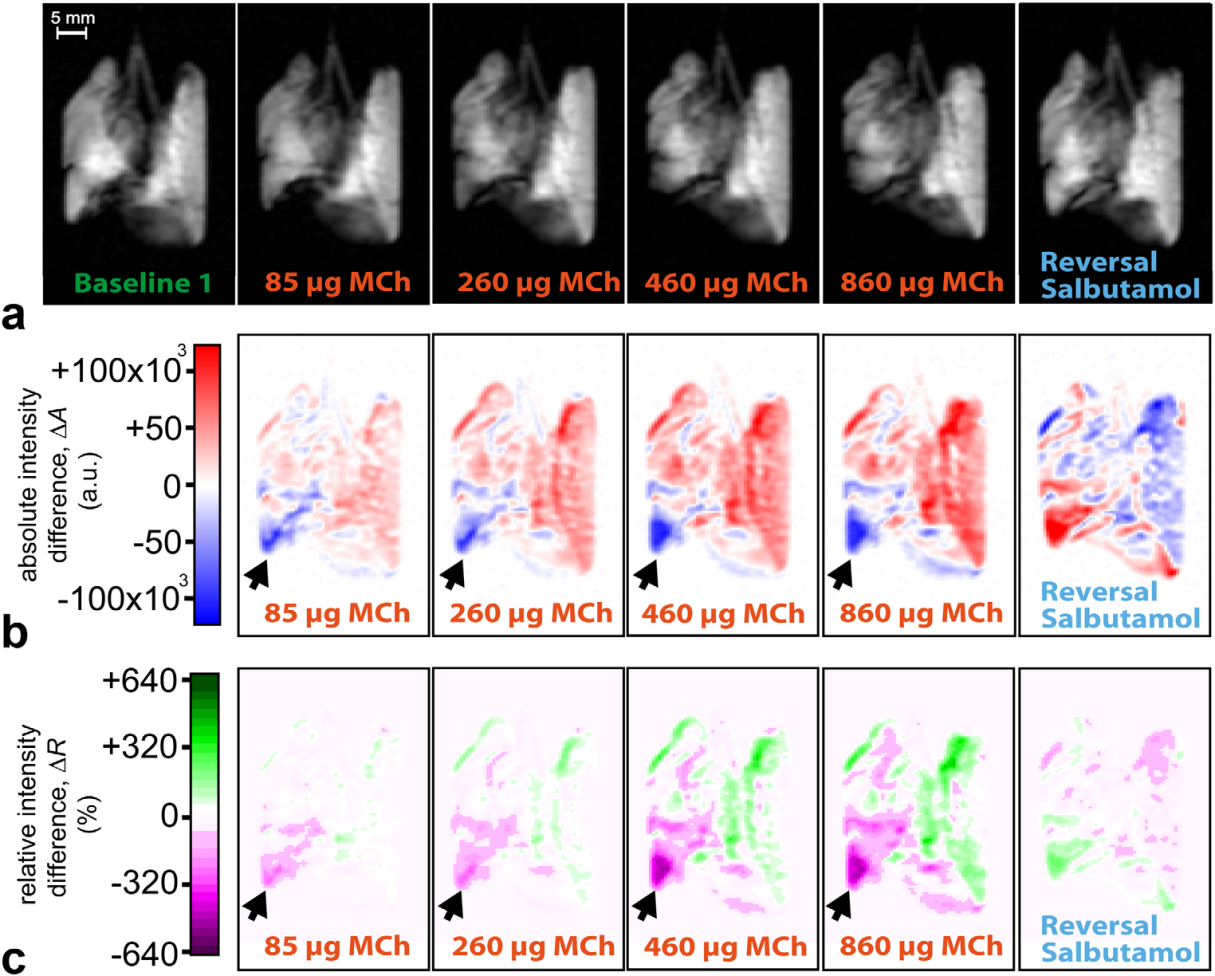
Image dataset from a control lung (C.1) with accompanying maps displaying changes in regional hp ^129^Xe distribution. **a**: Nonslice selective VFA FLASH image data from lung C.1 on cumulative dosages of MCh as indicated and on reversal with salbutamol and Hartmann's solution as displayed in Figure 3a–ii). **b**: Absolute intensity difference maps with difference between baseline image and increasing dosages of MCh and on reversal. Red indicates an increase in absolute hp gas signal intensity while blue indicates a reduction. **c**: Relative intensity difference maps (see Eq. [3]) indicating regional deviation of the inhaled gas between MCh challenge and baseline. Green indicates an increase in the regional share of inhaled gas with magenta a decrease compared with the global average. Note the reduction in the hp ^129^Xe distribution seen on the difference maps at the right caudal lobe (arrow) where there is a progressive reduction in signal intensity and the fraction of inhaled hp gas until it becomes visually apparent on the spin density VFA FLASH image in (a). Other lung regions show an increase in signal intensity and fractional hp gas inhalation. This basal region then permits hp gas entry on reversal with other lung regions demonstrating a reduction in signal intensity and fraction of inhaled gas. The full set of absolute intensity difference and relative intensity difference maps in lung C.1 after each dose of MCh are displayed in Appendix 2 in the Supporting Information.

**Figure 7:**
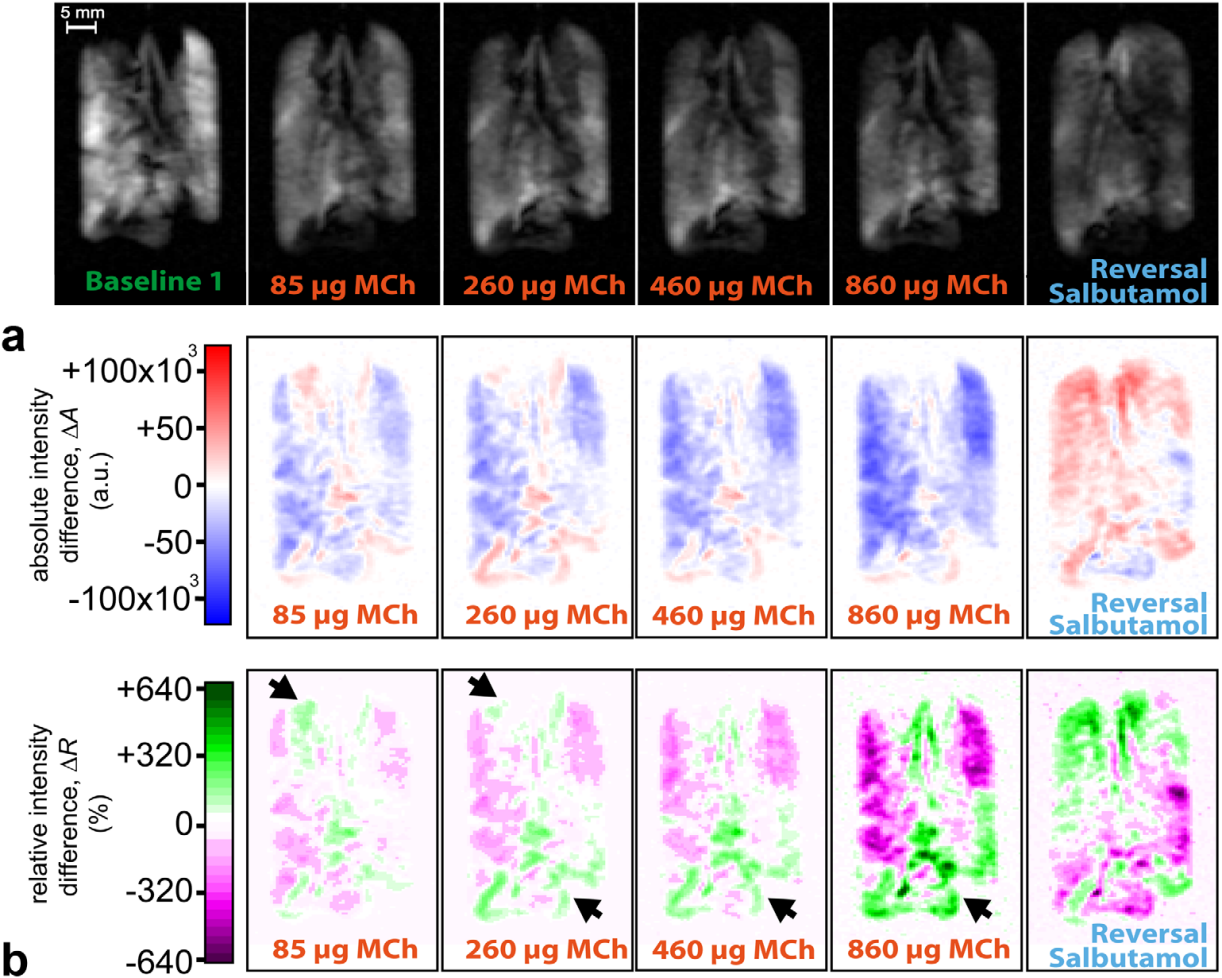
Image data from an OVA challenged lung (OVA.1) with accompanying maps displaying changes in regional hp ^129^Xe distribution. **a**: Nonslice selective VFA FLASH image data from lung OVA.1 on cumulative dosages of MCh (indicated) and on reversal with salbutamol and Hartmann's solution as displayed in Fig. 3b‐ii). **b**: Absolute intensity difference maps and (c) relative intensity difference maps indicating regional deviation of the inhaled gas from the mean inhalation as described in Figure 6 (Eq. [3]). Note the largely global reduction in the hp ^129^Xe inhalation seen on the absolute intensity difference maps across the whole lung on increasing MCh dosages, with small regions of increased signal intensity at the base of the lung and both apices. However, comparing the changes of the relative hp gas signal in (**c**) it is clear that these regions (arrows) experience a smaller reduction as compared with other lung regions, with a larger fraction of the global volume being contained within these regions. On reversal, hp gas distribution shows a global increase with fractional changes in hp ^129^Xe inhalation that are the opposite of that seen during the MCh challenges. The full set of absolute intensity difference maps and relative difference maps in lung OVA.1 after each dose of MCh are displayed in Appendix 2 in the Supporting Information.

The hp ^129^Xe MR images in Figures 6a and 7a provide some insights into regional inhalation pattern changes; however, a clearer picture emerges through two methods of data analysis:

- Maps displaying the absolute intensity difference, $\Delta A_{i}$ (Eq. [2]), between baseline images and images acquired on increasing MCh dosages are shown in Figures 6b and 7b, alongside difference maps acquired on salbutamol reversal. These maps depict regional changes in absolute hp ^129^Xe inhalation that may be above and below hp ^129^Xe inhalation in the baseline image.
- Relative intensity difference maps, $\Delta R_{i}$ (Eq. [3]), are shown in Figures 6c and 7c. Note that $\Delta R_{i}$ can assume values below negative 100% because baseline image voxels may have relative intensities above 100% of the mean value.

Using the absolute intensity difference maps, shown in Figure 6b, ventilation defects were detected before they became visually apparent on the spin density VFA FLASH images in Figure 6a. However, the drop in global intensity dominates the absolute intensity difference maps for the OVA challenged lung in Figure 7b thereby masking the regional changes in relative hp ^129^Xe inhalation. The gas redistribution can, in principle, be quantified using regional analysis of the differences in lung response between apical and basal lung lobes as reported in Appendix 3 in the Supporting Information. However, the regional redistribution of the total gas volume within the lung becomes visually apparent using the relative intensity difference maps in Figure 7c.

**Figure 8:**
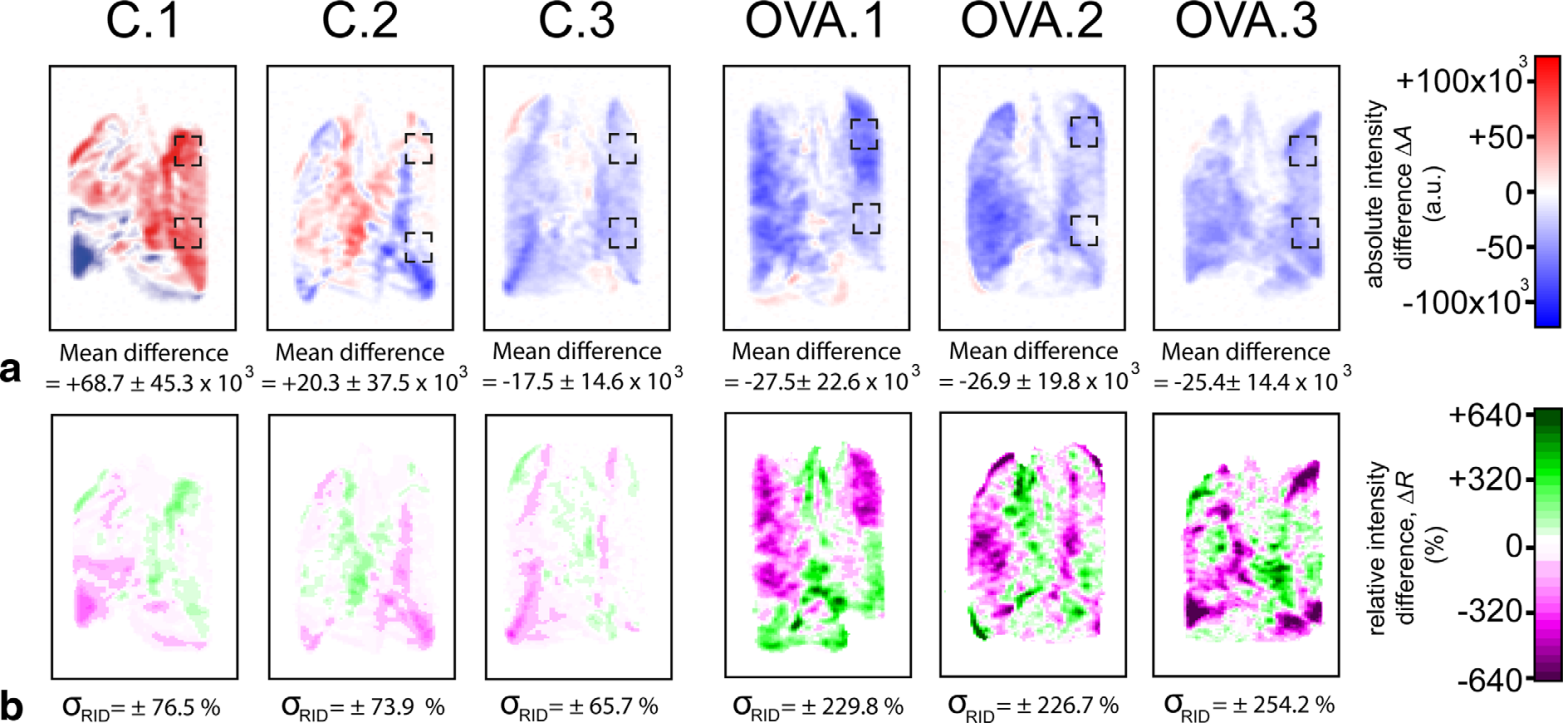
Absolute and relative intensity difference maps acquired from all imaged control and 500‐μg OVA challenged lungs. Columns contain difference maps from the same lung as indicated in the figure. (**a**) Absolute intensity difference map and (b) map indicating the regional deviation in hp gas volume from the mean of value after 860‐μg cumulative dosage MCh as seen in Figures 6 and 7. There is a global reduction in absolute signal intensity seen in C.3 and all the OVA challenged lungs after 860 μg of MCh with a notable reduction in voxel signal intensities quantified by the “mean difference”, i.e., the mean over all voxel values of absolute intensity difference (a). The hp gas is, however, seen to redistribute between different lung regions as seen on the maps in (**b**) with all lungs (control and OVA) displaying a large distribution of regional responses as indicated by the wide standard deviations of the relative intensity differences ( $\sigma_{RID}$), greater in the OVA treated lungs. Note the regions of interest (dashed square boxes) indicating locations selected for comparison of regional lung responses in Appendix 3 in the Supporting Material.

Figures 8a and 8b depict the absolute and relative intensity difference maps, respectively, for all control and 500 μg OVA challenged lungs after a cumulative MCh dosage of 860 μg. The control lungs C.1 and C.2 show noticeable redistribution of hp gas compared with the baseline as seen on the absolute and relative intensity difference maps. Control lung C.3, however, also demonstrates a noticeable drop in the absolute signal intensities across the image series that is largely due to the drop seen during initial fluid administration. By comparison, all the OVA challenged lungs show a decrease in absolute signal intensities across the series. Furthermore, as can be taken from the scale in Figure 8b, the relative intensity differences for the OVA model lungs increase compared with that of the control lungs. This increase can be quantified by the standard deviation of the relative intensity difference ( $\sigma_{RID}$) that was approximately three‐fold elevated for the OVA model compared with $\sigma_{RID}$ of the control lungs due to the increased heterogeneity of hp ^129^Xe distribution.

## DISCUSSION

### Changes in hp ^129^Xe Inhalation Patterns Detected by Ex Vivo Hyperpolarized ^129^Xe MRI

Ex vivo hyperpolarized ^129^Xe MRI has been shown to allow for imaging of dynamic changes in hp ^129^Xe inhalation in a rat OVA model of human asthma. The hp gas inhalation protocol and MCh administration demonstrated significant bronchoconstriction. The overall reduction in inhaled hp gas volumes is greater in OVA treated lungs compared with control lungs as noted by the decrease in global signal intensity, GS, (*P* = 0.03). With an observed >50% reduction in whole lung signal intensities (approximating to a 50% reduction in inhaled gas volumes), these data may, therefore, have relevance in severe to life‐threatening asthma. Reversal was not noticeable in measurements of the normalized global signal intensity, GS, (Eq. [1]); however, reversal affected the regional gas distribution.

Using the absolute and relative intensity difference maps, regional gas distribution changes were seen before they became visually apparent on hp ^129^Xe spin density images. While the absolute intensity difference maps of the OVA treated lungs are dominated by the drop in global signal intensity, GS, the maps of relative intensity difference, as defined by Eq. [3], reveal the underlying changes regional hp ^129^Xe inhalation (Fig. 7c). Furthermore, an approximately three‐fold increased standard deviation of the relative intensity difference, $\sigma_{RID}$, in the OVA model lungs (Fig. 8b) reveals an increase in the regional heterogeneity of the hp ^129^Xe inhalation (relative to GS) compared with the control lungs. Note that this work builds on testing of the ex vivo delivery protocol in otherwise unrelated studies (see Appendix 4 in the Supporting Information for an example) where it was ensured that repeated inflation and deflation of the rat lung after transport on the times scales indicated (over 5 hours) would not affect results or significantly impact on regional hp ^129^Xe inhalation patterns.

Chen et al. have previously studied the effect of high doses of intravenous (i.v.) MCh (30 μg) on healthy Sprague‐Dawley rats using hp ^3^He MRI 24. It was hypothesized that there was a partitioning in the lung response where gas destined for lung regions with significant constriction was diverted to less constricted zones, with associated hyperinflation. The regional data contained within this work confirms this result where gas is seen to be increasingly diverted to less constricted regions during rising dosages of MCh. However, unlike in the live animal where the total dose of MCh is limited due to systemic concerns, much higher dosages can be administered repeatedly in the ex vivo model 24. Ex vivo hp ^129^Xe MR imaging in the current work was performed using constant pressure inhalation, similar to in vivo work by other groups 27, as this was found to be most sensitive to changes in hp gas inhalation and with less chance of lung rupture.

Note that image registration methods were not used in the current proof of principle work and, therefore, artifacts, particularly noticeable in airways and at the edge of lung fields, did occur. In earlier work, Driehuys et al demonstrated regional hp ^3^He MRI measurements of the lung parenchyma and on the major airways 55. This enabled a simple pixel counting method to quantify variation in localized responses between the two tissues in OVA challenged mice post MCh. The hp ^3^He signal intensity from a volume within the trachea was used by Mistry et al 52 for normalization of each voxel value. Images were thereby less dependent on total image signal intensity and difference maps, generated between baseline images and on increasing dosages of MCh, enabled visualization of regions with reductions or increases in hp gas distribution. Furthermore, quantification of changes in airway diameters was also possible. However, changes in tracheal diameter my cause problems for usage of tracheal signal intensity for image normalization, while in the current work normalization was performed to the less variable total signal intensity (Eq. [3]).

Deninger et al described the concept of “fractional ventilation”, defined as the volume of “new” (hyperpolarized) gas that replaces “old” (nonhyperpolarized) gas on subsequent ventilations 56. This concept was subsequently applied to an *Aspergillus fumigatus* mouse model of asthma 25 demonstrating a significant reduction in fractional ventilation values across asthmatic mouse lungs with considerable heterogeneity between different lung regions 27, 57. Fractional ventilation was not used for the ex vivo model reported in this proof of concept work that used a single breath‐hold without data averaging; however, the ex vivo concept could easily be extended to accommodate fractional ventilation studies if so desired.

Ex vivo whole lung models have been studied for several years. Initially, these measurements were limited to studies of ventilation parameters in healthy lung tissue 32, 58, 59, 60. Later developments using more sophisticated models studied dynamic ventilatory changes in healthy rodent lungs 34, 42. There are several similarities of the experimental setup contained within this work, including the use of a negative pressure differential upon the ventilation chamber that acts as artificial pleural cavity to enable “active” inhalation and the delivery of physiological perfusate and bronchoactive substances by means of the pulmonary circulation. Necessary simplifications were, however, required to allow for hp ^129^Xe MRI including the absence of recirculation of warmed perfusate. Furthermore, like other ex vivo whole lung models, it should be noted that the artificial pleural cavity is usually an arbitrary shape, such as a cylinder in the case of this work, which may result in some nonphysiological lung deformation and gas redistribution.

Previously, it has been noted that lung responses using healthy excised ventilated and perfused mouse lungs have closely matched those seen using PCLS models 34. However, several groups have found it difficult to detect differences in AHR between control and OVA treated small animal lung tissue using PCLS methods 28, 61. Whether this is due to low numbers of large airways included in each lung slice, the absence of other lung related changes such as increased airway resistance or possibly the technical issues surrounding the nonphysiological delivery of bronchoprovocative compounds is unknown. Furthermore, an important role for the circulatory system in the development of AHR has been described 62. The absence of the circulatory system in PCLS models may help explain why differences in AHR have not been detected using this model. It should be noted that use of whole ex vivo lungs suffers from none of these limitations.

As some final considerations, it should be noted that significant differences in AHR to MCh were detected between control and OVA lungs using relatively low numbers of animals (n = 3 group sizes in this work) as compared to other previously reported in vivo plethysmographic 21 and hp ^129^Xe methods 26. Although the necessity for animal culling to perform the ex vivo experiments precludes longitudinal studies, it is possible that this methodology could reduce animal numbers if serial measurements are not required. Furthermore, animal suffering with the ex vivo model could potentially be further reduced by using healthy ex vivo lungs treated with sera from small numbers of separately sensitized animals 63, allowing for rapid model generation and throughput investigation which is not possible using in vivo methods.

## CONCLUSIONS

This is the first work investigating a whole organ ex vivo animal model of the airway hyper‐responsiveness found in human asthma using hp ^129^Xe MRI methodology. Changes in both whole lung and regional measurements of hp ^129^Xe inhalation were detected, demonstrating significant differences in the normalized global signal intensities between control and OVA challenged animals using small sample sizes (*P* < 0.05). OVA models were affected by reduced overall hp ^129^Xe inhalation at small MCh dosages that was not observed in control lungs. However, control lungs exhibited localized alterations in the regional gas distribution, visible in absolute intensity difference maps that may be caused by the diversion of gas between different lung regions. Relative intensity difference maps revealed that regional heterogeneity was also present in the inhalation patterns seen in the OVA sensitized lungs after MCh challenge and the relative heterogeneity in the OVA model, quantified by the standard deviation of the relative intensity differences ( $\sigma_{RID}$), was increased by a factor of 3 compared with the control. The relatively straightforward ex vivo hp noble gas imaging arrangement and the image analysis methodology may be useful as an adjunct to current investigational techniques and may further encourage the use of ex vivo lung tissue.

## Supporting information


**APPENDIX 1**. Complete image datasets demonstrating dynamic changes in hp ^129^Xe gas distribution on increasing dosages of methacholine (MCh).
**APPENDIX 2**. Complete dataset demonstrating changes in regional hp ^129^Xe distribution on increasing dosages of methacholine.
**APPENDIX 3**. Quantification of changes in hp ^129^Xe gas distribution on increasing dosages MCh from selected lung regions.
**APPENDIX 4**. Example data demonstrating changes in hp ^129^Xe gas distribution during repeated inhalations over a similar time course.
