# Supplementary material for: Investigating lung responses with functional hyperpolarized xenon‐129 MRI in an ex vivo rat model of asthma
Source: Magn Reson Med. 2015 Oct 28;76(4):1224–35. doi: 10.1002/mrm.26003 (PMC5026173; doi:10.1002/mrm.26003)
Supplement: Supplementary file 1 — APPENDIX 1. Complete image datasets demonstrating dynamic changes in hp 129Xe gas distribution on increasing dosages of methacholine (MCh). APPENDIX 2. Complete dataset demonstrating changes in regional hp 129Xe distribution on increasing dosages of methacholine. APPENDIX 3. Quantification of changes in hp 129Xe gas distribution on increasing dosages MCh from selected lung regions. APPENDIX 4. Example data demonstrating changes in hp 129Xe gas distribution during repeated inhalations over a similar time course. [file MRM-76-1224-s001.docx]

Supporting Information

Appendix 1- Complete image datasets demonstrating dynamic changes in hp ^129^Xe gas distribution on increasing dosages of methacholine (MCh).

The full image datasets acquired from the control and OVA challenged animals displayed in Fig. 3 are given in Fig. S1 and S2, where changes in hp ^129^Xe distribution that is characteristic of increasing doses of MCh are demonstrated. Images were taken at: baseline (2 repeat measurements); after a 4.5 mL bolus of Hartmann’s; on increasing doses of MCh (10, 25, 50, 100, 200 and 400 μg); and on subsequent reversal with flushes of varying volumes of Hartmann’s solution and up to 1500 μg of salbutamol. The presence of ventilation defects (regions with absent hp gas signal) was noticeable in the control lung (Fig. S1) after 200 μg MCh, i.e. 460 μg cumulative MCh dose and on 400 μg MCh (860 μg cumulative MCh dose), with complete reversal seen after flushing with 29.0 mL Hartmann’s solution and 1000 μg of salbutamol. The OVA treated lung in Fig. S2 demonstrated significant heterogeneity in hp ^129^Xe inhalation at baseline. The OVA treated lung then displays a noticeable reduction in signal intensity on the 50 μg dose of MCh with further significant reductions until 400 μg MCh. After the cumulative dosage of 860 μg of MCh, subsequent reversal with 1500 μg salbutamol and 49.0 mL Hartmann’s solution is seen in the OVA treated lung.

**
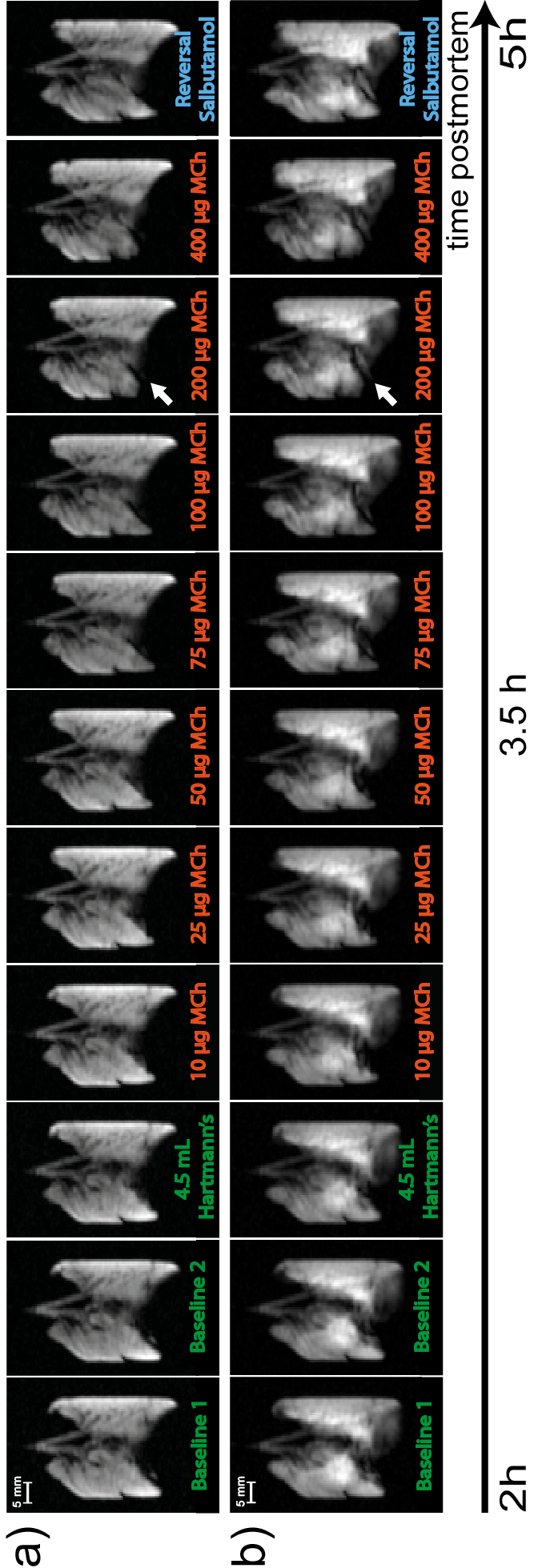
**

**Figure S1. Full VFA FLASH image dataset from control lung at baseline and on increasing doses methacholine (MCh) with subsequent reversal.** (a) Slice selective and (b) non-slice selective image data from control rat C.1. Window levels are uniform for MR signal intensity within both image datasets. Note the close correspondence between baseline images and after flushing with 4.5 mL Hartmann’s solution. Noticeable ventilation defect in right lower lobe after 200 μg MCh (white arrows) with subsequent reversal after 1000 μg salbutamol and 29.0 mL Hartmann’s solution.


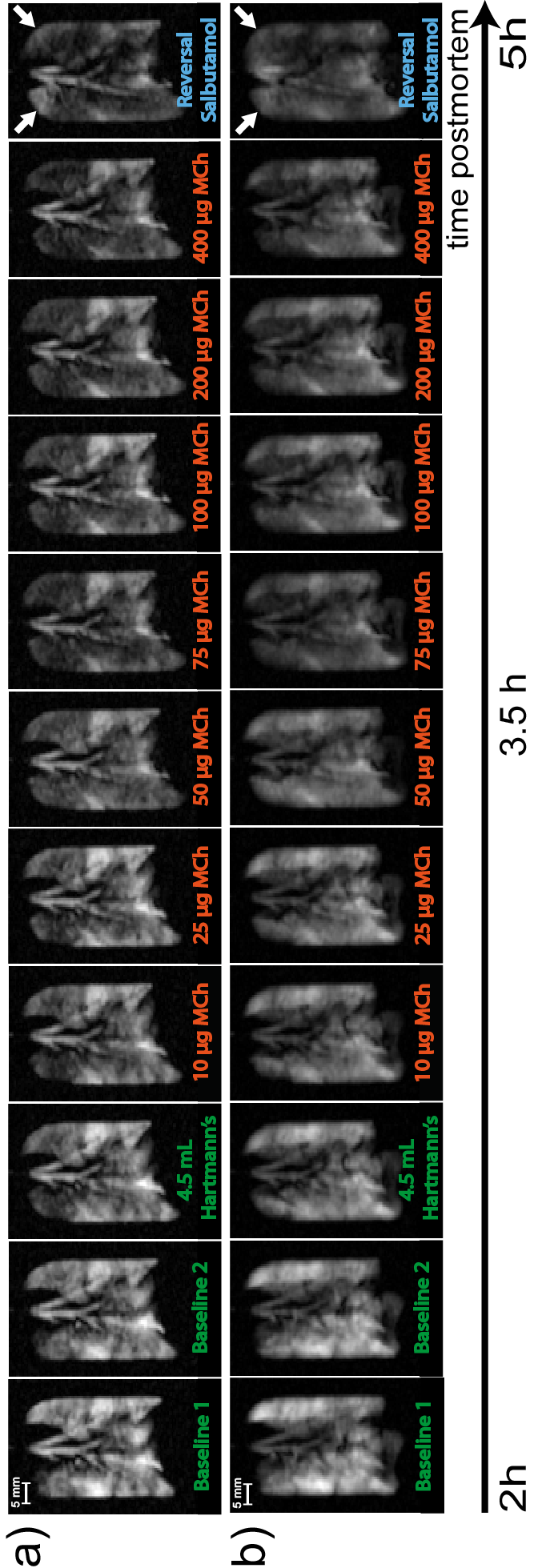


**Figure S2. Full VFA FLASH image dataset from an ovalbumin (OVA) lung at baseline and on increasing doses methacholine (MCh) with subsequent reversal.** (a) Slice selective and (b) non-slice selective image data from OVA challenged rat OVA.1. Window levels are once again uniform for MR signal intensity within both image datasets. Note the significant heterogeneity in hp gas distribution on the baseline images and a small reduction in signal intensity in the right cranial lobe after flushing with 4.5 mL Hartmann’s solution. There is a noticeable reduction in signal intensity on the 50 μg dose of MCh with further significant reductions until 400 μg MCh. Subsequent reversal with 1500 μg salbutamol and 49.0 mL Hartmann’s solution when signal increases in the right cranial lobe and the upper portion of the left lobe (white arrows).

Appendix 2- Complete dataset demonstrating changes in regional hp ^129^Xe distribution on increasing dosages of methacholine.


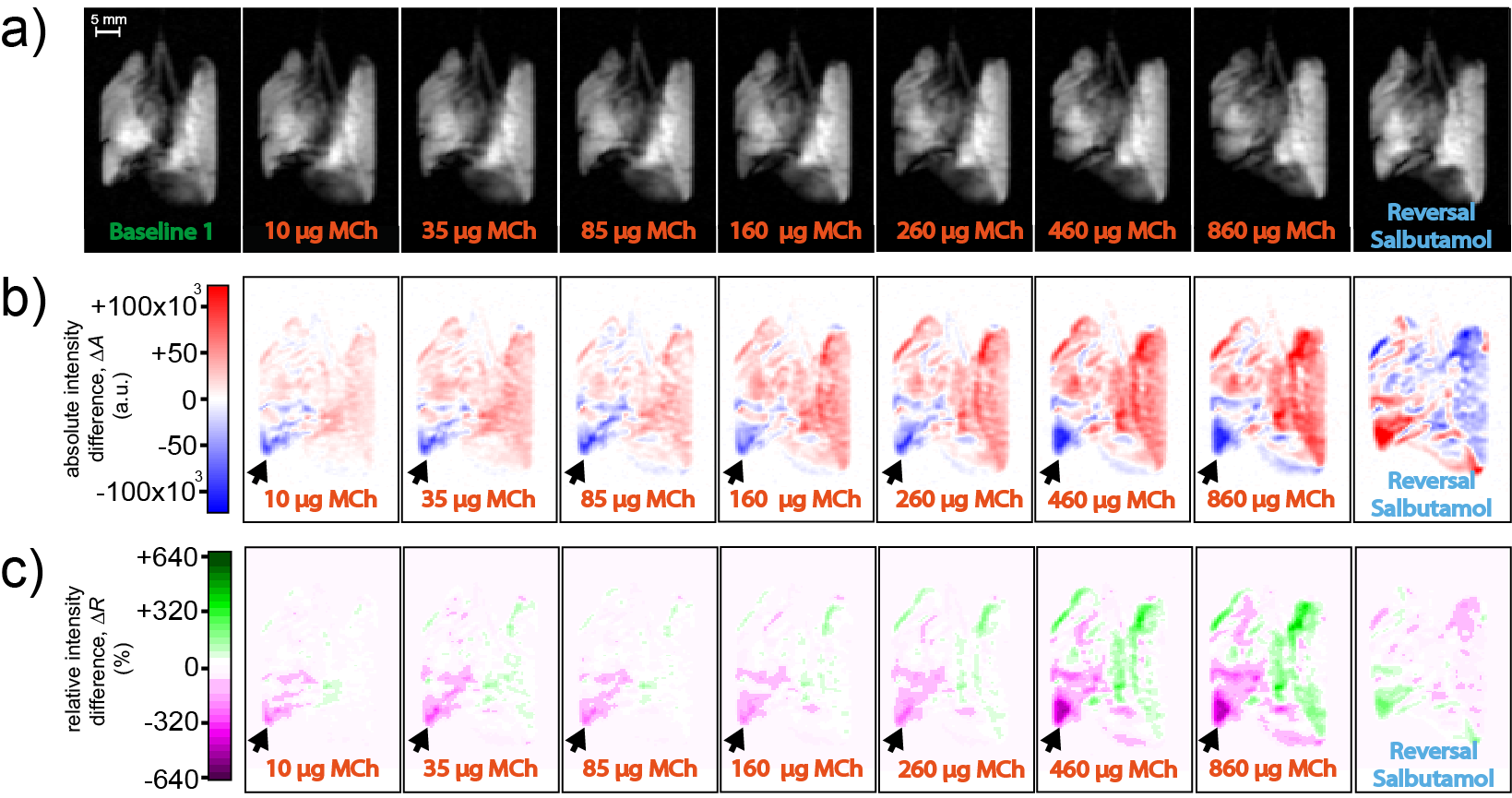


**Figure S3. Image data from a control lung with accompanying maps displaying changes in hp ^129^Xe distribution after each delivered dose of methacholine (MCh).** (a) Non-slice selective VFA FLASH image data from lung C.1 on cumulative dosages of MCh (indicated) and on reversal with flushes of 29 mL Hartmann’s solution and 1000 μg salbutamol. (b) Absolute difference maps with difference between baseline image and increasing dosages of MCh and on reversal. Red indicates an increase in absolute hp gas signal intensity while blue indicates a reduction. (c) Difference maps indicating regional deviation of the inhaled gas from the mean inhalation after each dosage of MCh and baseline. Green indicates an increase in the regional fraction of inhaled gas with magenta a decrease. Note the reduction in ventilation seen on the difference maps at the right caudal lobe (arrows) where there is a progressive reduction in signal intensity and the fraction of inhaled hp gas between 10 – 460 μg of MCh when it becomes visually apparent on the spin density VFA FLASH image in (a). Other lung regions show an increase in signal intensity and fractional hp gas inhalation. This region then permits hp gas entry on reversal with other lung regions demonstrating a reduction in signal intensity and fraction of inhaled gas.


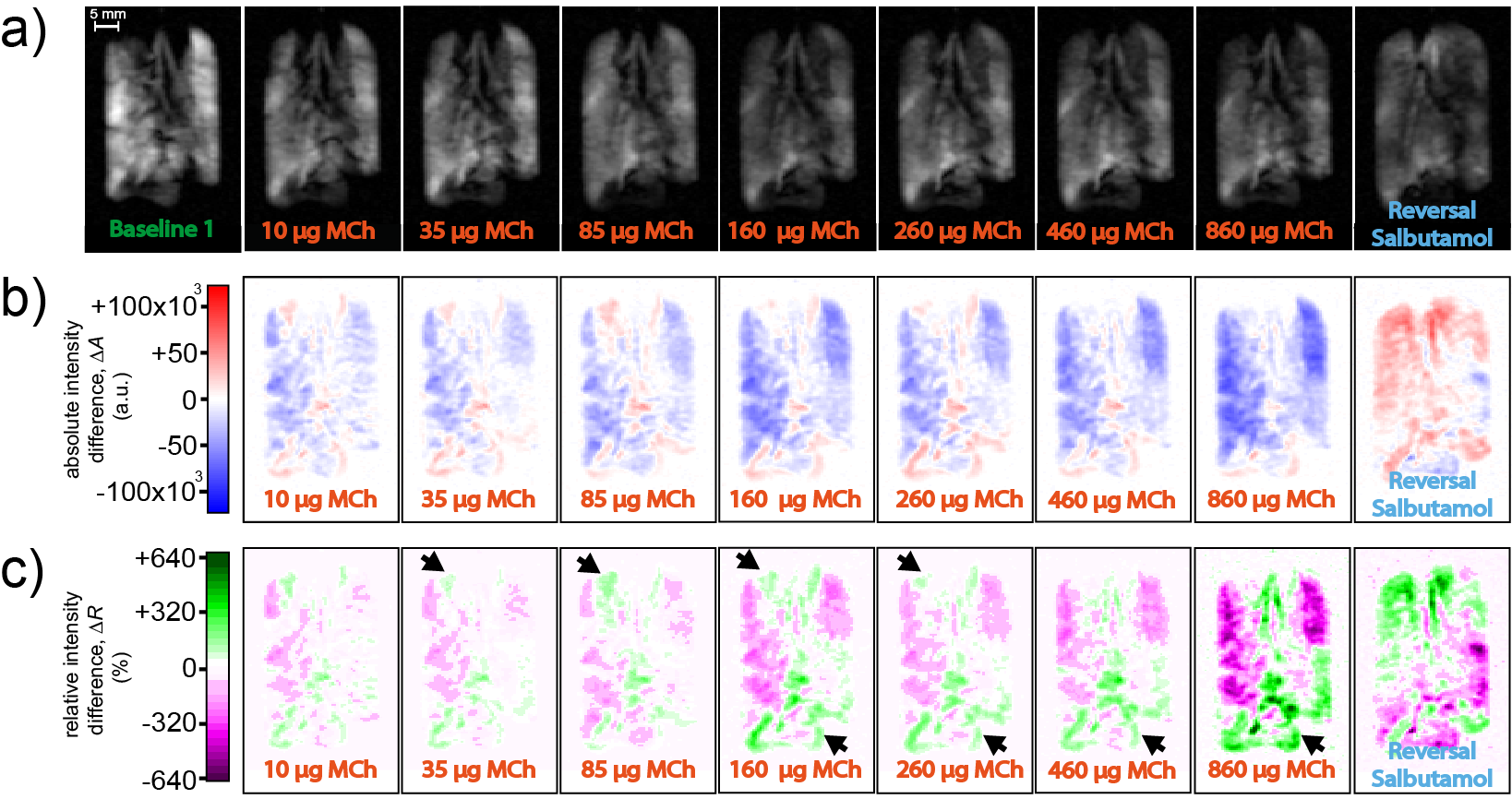


**Figure S4. Image data from an OVA challenged lung with accompanying maps displaying changes in hp ^129^Xe distribution after each delivered dose of methacholine (MCh).** (a) Non-slice selective VFA FLASH image data from lung OVA.1 on cumulative dosages of MCh (indicated) and on reversal with flushes of 49 mL Hartmann’s solution and 1500 μg salbutamol. (b) Absolute difference maps and (c) maps indicating regional deviation of the inhaled gas from the mean inhalation as described in Fig. S3. Note the largely global reduction in ventilation seen on the absolute difference maps across the whole lung between 10 – 860 μg with small regions of increased signal intensity at the base of the lung and both apices. However comparing the fraction of hp gas signal in (c) it is clear that these regions (arrows) contain increasing volumes of the total the total gas inhaled. On reversal, hp gas distribution shows a global increase with small reductions in the regions indicated (arrow) with the change in fractional distribution opposite to that seen during the MCh challenges.

Appendix 3- Quantification of changes in hp ^129^Xe gas distribution on increasing dosages MCh from selected lung regions.

As a method to study the regional MCh responses, 8 × 8 voxel regions of interest (ROIs) were selected from the upper and lower left lung lobe (as indicated in Fig. 8 in the main text) with the reduction in regional mean normalized signal intensity compared on increasing MCh dosages (see Fig. S5). Among the control lungs, C.2 showed a difference in response between the basal and apical regions (Fig. S5a – b). The 500 μg OVA challenged lungs (Fig. S5c – d) however demonstrated a greater response to MCh in the apical regions of the left lobe compared to the basal section of the lobe in all imaged lungs.


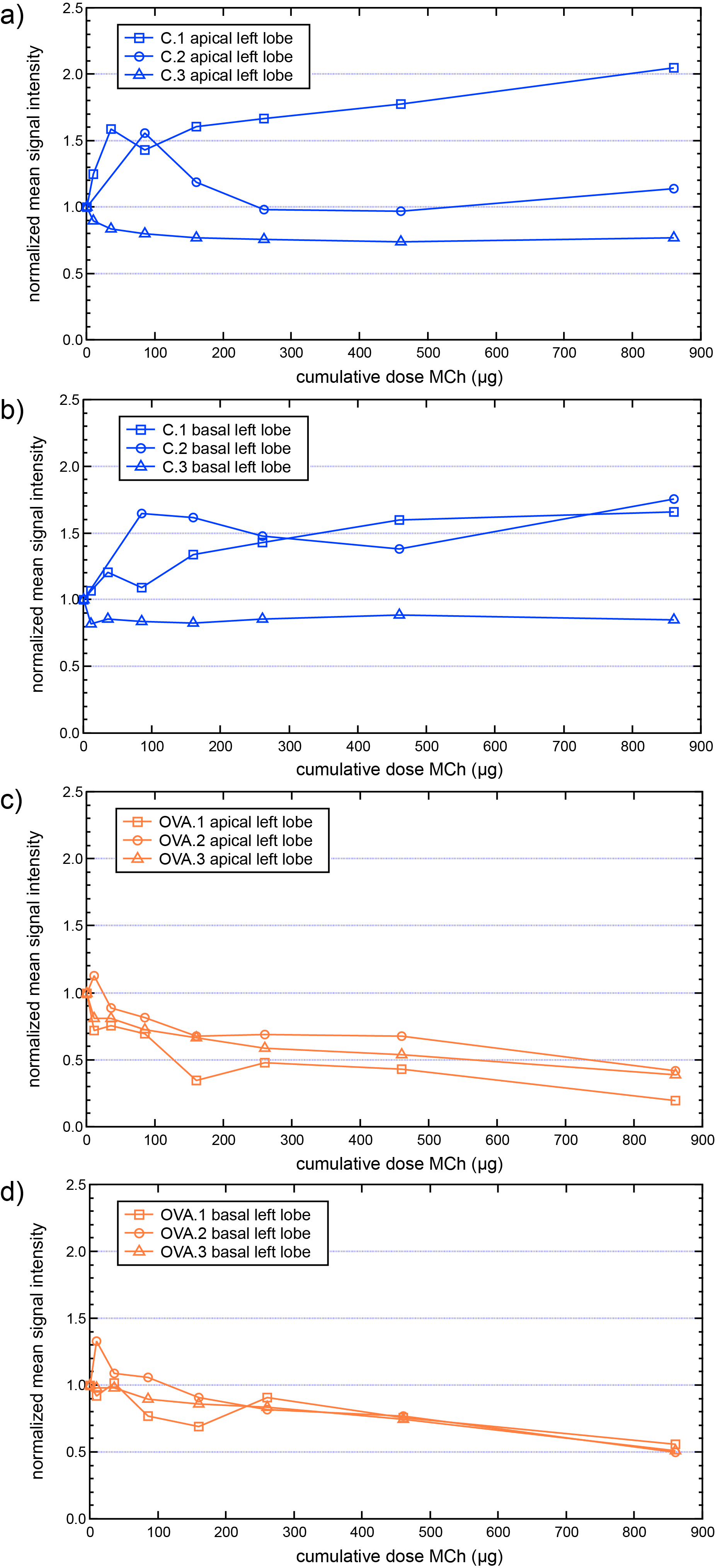


**Figure. S5. Regional differences in mean normalized signal intensity in control and 500 μg OVA challenged lungs.** Measurements taken for 8 × 8 region of interest (ROI) in apical and basal locations of left lobes of non-slice selective image data indicated in Fig. 8a Apical and basal left lobe measurements from the hp ^129^Xe imaged control lungs. Regional differences in response to MCh can be seen in lung C.2 where the basal region of the left lobe shows a large increase in mean normalized signal intensity while the apical section shows an initial increase followed by decrease and subsequent plateau. (c - d) Similar measurements as in (a – b) from the hp ^129^Xe imaged 500 μg OVA challenged lungs. There is a greater response to MCh in the apical region of the left lobe compared to the basal section of the lobe in all OVA challenged lungs imaged.

Appendix 4- Example data demonstrating changes in hp ^129^Xe gas distribution during repeated inhalations over a similar time course.


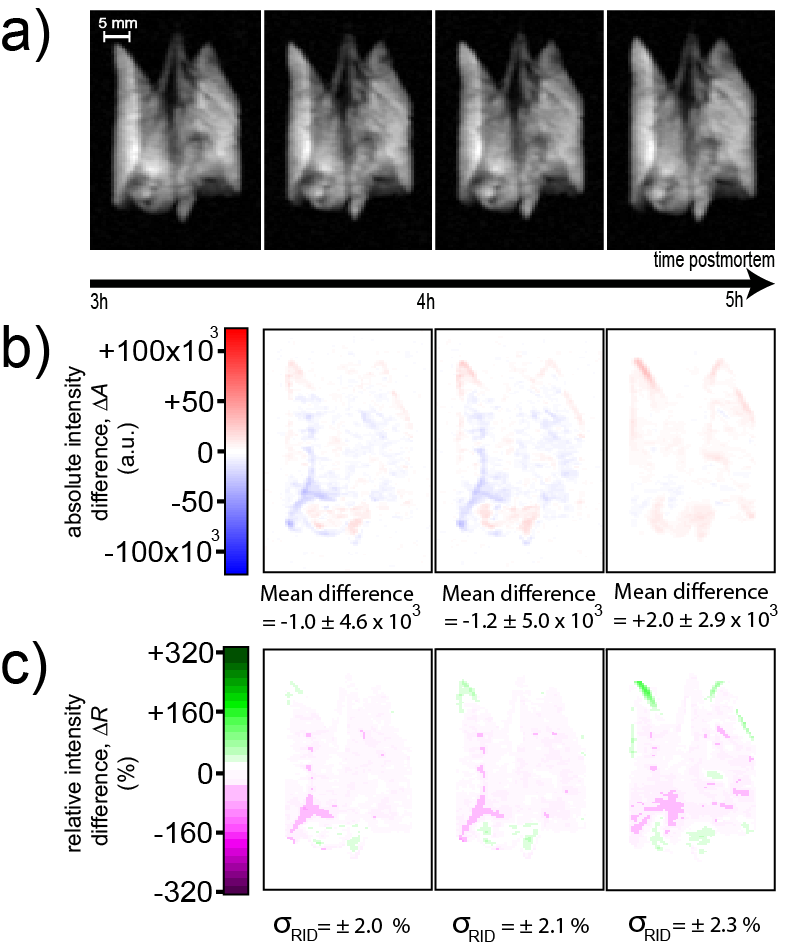


**Figure. S6. Example healthy *ex vivo* lung with repeated inhalations over timeframe of 3 – 5 hours post-mortem with respective difference maps.** (a) Non-slice selective VFA FLASH image data acquired at 30 minute intervals. (b) Absolute and (c) maps indicating regional deviation of the inhaled gas from the mean inhalation as described in Fig. S3 and Fig. 8 with the standard deviations of the relative intensity differences ($\sigma_{RID}$) reported in (c). Data acquired from healthy Brown-Norway rat (weight 278 g at time of death). Preparation for *ex vivo* lung imaging performed as detailed in the Methods section with similar ventilation and hp ^129^Xe MRI parameters. Note that there is some variation in the absolute difference maps, but that this is far less than is seen in MCh treated lungs in Fig. 8. Furthermore, this could be improved with finer control of inhalation volumes and image registration methods.
